# Measuring the Development of Therapeutic-Decision-Making Skills by Practicing Pharmacists Undertaking a University-Based Postgraduate Clinical Qualification at Distance

**DOI:** 10.3390/pharmacy8020083

**Published:** 2020-05-11

**Authors:** Daniel F. B. Wright, Stephen B. Duffull, Kyle J. Wilby, Aynsley K. Peterson, Megan G. Anakin

**Affiliations:** 1 School of Pharmacy, University of Otago, 9054 Dunedin, New Zealand; stephen.duffull@otago.ac.nz (S.B.D.); kyle.wilby@otago.ac.nz (K.J.W.); aynsley.peterson@otago.ac.nz (A.K.P.); 2 Education Unit, Dunedin School of Medicine, University of Otago, 9054 Dunedin, New Zealand; megan.anakin@otago.ac.nz

**Keywords:** pharmacy education, continuing professional development, therapeutic decision-making

## Abstract

(1) **Background:** The processes and skills required to make decisions about drug therapy have been termed “therapeutic decision-making” in pharmacy practice. The aim of this study was to evaluate a tool constructed to measure the development of therapeutic-decision-making skills by practicing pharmacists undertaking a university-based continuing professional development program. (2) **Methods:** A pre- and post-intervention crossover study design was used to investigate the qualitative and quantitative features of practicing pharmacists’ responses to two clinical vignettes designed to measure the development of therapeutic-decision-making skills. The vignettes were assigned a score using a five-point scale and compared pre- and post-intervention. (3) **Results:** There was a median increase in score of 2 units on the five-point scale in the post-intervention scores compared to pre-intervention (*p* < 0.0001). (4) **Conclusions:** The results were interpreted to suggest that the participants’ responses to the vignettes are a reasonable measure of student learning. Therefore, we infer that the teaching and learning intervention successfully enabled the development of therapeutic-decision-making skills by practicing pharmacists.

## 1. Introduction

“… *pharmacist training would need to deliver resilient, accountable, autonomous, patient-centred, prescribing pharmacists*.”

- Health Education England. Advancing pharmacy education and training: a review 2019

A simple search of the internet with any browser using a combination of terms like “pharmacy practice” or “pharmacy profession” and “evolution” or “change” will bring up a large number of recent publications and documents all saying basically the same thing: the pharmacy profession is in a period of rapid change. This evolution has been a long time coming. Commentators over the past 60 years like Brodie [1] and the Millis Commission [2], as well as the seminal work of Hepler and Strand [3,4], envisioned a pharmacy profession focused primarily on patient-facing clinical services, rather than medicines supply. The recent push to make this a reality has been driven by several factors, notably changes in regulatory frameworks worldwide to allow for an expanded scope of practice for pharmacists [5] and changes to the funding environment to include clinical pharmacy services [6]. 

The quote above from Health Education England encapsulates a general feeling across the pharmacy sector; that educators need to keep pace with the changing health care landscape to deliver a pharmacy workforce that is fit for purpose. Moving forward, pharmacists must have transferable skills and knowledge that will prepare them for roles with an increasing focus on the delivery of clinical services, including prescribing. These roles will inevitably involve pharmacists taking direct responsibility for therapy decisions, something that has not traditionally been a feature of pharmacy professional identity. In addition, pharmacists will need skills to aid decision making and to untangle priorities in complex patients with multiple comorbidities. Continuing professional development (CPD) for pharmacists has a critical role to play in this changing environment. Not only does CPD allow new pharmacists to expand their foundational skills during early career development, but CPD is also an important means of upskilling pharmacists already in practice who were trained at a time when patient-facing clinical services were less prevalent. 

The processes and skills required to make decisions about drug therapy have been termed “therapeutic decision-making” in pharmacy practice [7,8,9] and “management decision-making” in medicine [10,11]. Decision making is recognized as an important component of professional pharmacy competency, however, the explicit teaching of therapeutic decision-making is underdeveloped in both pharmacy and medicine education. Here, we distinguish decision making about drug therapy from the processes associated with medical diagnostics. The latter is a particular focus of medical education. It has been suggested that decisions about managing drug therapy will require different skills and processes than those required for diagnosis [10,11]. 

Our group has previously proposed a teaching model for therapeutic decision-making [7] aligned with a philosophical framework for pharmacy practice [12]. The model builds on the seminal publications of Hepler and Strand [3,4] as well as Bryant [13], Sexton et al. [14] and the pharmacist patient care process (PPCP) framework [15], and is explicitly designed to allow students to surface the tacit cognitive processes required to work through therapeutic decisions. The model for therapeutic decision-making has been implemented in undergraduate and post-registration clinical qualifications for practicing pharmacists at the University of Otago in New Zealand. It is currently unclear is what impact the teaching has on the development of therapeutic-decision-making skills by practicing pharmacists in a continuing professional development context. 

The aim of this study was to evaluate the utility of a vignette to measure the development of therapeutic-decision-making skills by practicing pharmacists who were undertaking a university-based postgraduate clinical program at distance.

## 2. Materials and Methods 

### 2.1. Study Context and Participants

This study took place at the School of Pharmacy at the University of Otago, New Zealand. The subjects were practicing pharmacists enrolled in a part-time post-registration clinical qualification delivered at distance (the Postgraduate Certificate in Pharmacy endorsed in Medicines Optimization). The clinical qualification ran over two semesters. The teaching intervention and study described here was part of a 12 week course called “Patient Centered Care” (PHCY511). The therapeutic-decision-making module within this course ran over 4 weeks. Note that in New Zealand, many practicing pharmacists return to university for continued training post-registration. 

### 2.2. Study Overview

In brief, the study involved three steps: (1) prior to any teaching about therapeutic decision-making, students were asked to read a brief clinical vignette and describe the processes they would undertake to make a decision about the patient’s drug therapy; (2) a teaching intervention about therapeutic decision-making over four weeks was undertaken; and (3) a second, post-intervention, clinical vignette was administered and students were again asked to describe the processes they would undertake to make a decision about the patient’s drug therapy. A mixed methods approach [16] was used to address the aim of this study. A pre- and post-intervention crossover study design was used to investigate the qualitative and quantitative features of the practicing pharmacists’ responses to the clinical vignettes that was designed to measure their therapeutic-decision-making skills. The study design was approved through departmental peer review and ethical approval was granted from the University of Otago Human Ethics Committee (D19/261). The significance of this study to Māori was discussed with the Ngai Tāhu Research Consultation Committee. This study was conducted in accordance with the Declaration of Helsinki [17] and all participants gave their informed consent to be involved in this study.

The therapeutic-decision-making teaching model used, the teaching and learning intervention, study instrument, study procedures and data analysis are described in detail below. 

### 2.3. The Teaching Model

The model for therapeutic decision-making includes four steps [7]. Step one is “information gathering” and may involve several components including: establishing a rationale for a decision, interpreting laboratory results, identifying drug-related problems, specifying treatment and patient-centered goals, assessing the patient physically and psycho-socially, reviewing relevant literature and considering patient factors that may impact drug therapies. Methods for prioritizing important clinical problems in complex patients are included in this step. Step two (“reasoning”) requires the collation and appraisal of the information gathered. This is contextualized in relation to the patient’s goals and the medical goals of therapy. Step three is “judgement” and may include an analysis of benefit-risk for available treatment options. The impact of each option on the treatment outcomes for the patient’s health, the patient’s family and other health care providers as well as the financial and social impacts are considered. Step four is enacting and monitoring the decision. The teaching model also includes a reflective component to allow students to unpack their decision-making.

### 2.4. Teaching and Learning Intervention

A four week teaching and learning module was conducted to introduce practicing pharmacists to the processes and skills involved in making therapeutic decisions. The module was preceded by an introductory in-person workshop which included interactive tutorials designed to provide learners with the background to therapeutic decision-making and why it is important for pharmacy practice. The tutorials were also used to present the structured model for therapeutic decision-making (above) and to introduce skills to untangle important clinical problems in complex, chronic-care patients with multiple comorbidities. Learners were introduced to a real-time patient simulation tool called SimPHARM [18] using demonstrations and a hands-on exercise. The structured model for learning therapeutic-decision-making skills was built into the SimPHARM environment and as the learners worked through a demonstration patient case they were asked to document the therapeutic decisions made. After the introductory workshop, the remainder of the teaching and learning for the course occurred at distance.

Over the four week module, the learners were asked to work through two patient cases, at week two and week four, using SimPHARM. The cases ran for about three to four days in real time. Learners documented their decisions and justifications in the SimPHARM environment. This documentation was reviewed by the course tutors who were also researchers in this study. For the first case, specific formative feedback was provided to individual learners in writing and then general feedback was given to the cohort via a video conference by the tutors. For the second case, learners documented their decisions and clinical justifications and were then asked to provide an extended explanation about one important decision they made about the case. A marking rubric was available to learners, and as a reflective exercise, they were asked to mark the second case. Learners submitted their score, along with their other documentation, for marking and feedback from their course tutors as part of the assessment procedure for the module.

### 2.5. Study Instrument (Vignettes)

**Figure 1:**
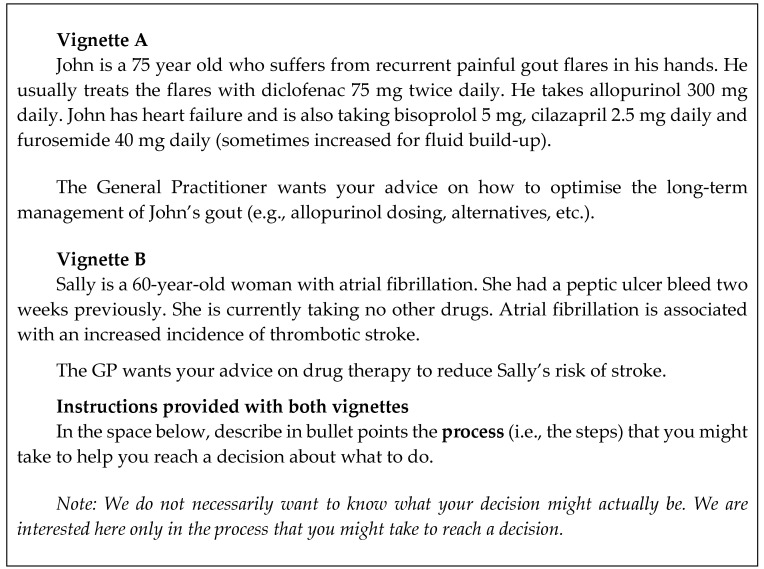
Vignettes with instructions used for data collection.

The data collection instrument designed for this study was a clinical vignette. The vignettes were written by the members of the research team with knowledge of clinical pharmacy practice and were worded to be familiar to pharmacists practicing in New Zealand. There were two vignettes written (A, B) that included a case scenario with prompts for written responses shown in Figure 1.

### 2.6. Data Collection

Data was collected at two time points: once at the beginning of the module prior to any teaching about therapeutic decision-making and again at the end of the teaching intervention after week 4 of the course. All learners enrolled in the course were invited to participate in the study by a member of research team who was not involved in the course to ensure learners did not feel coerced to participate by their teachers. The first data collection point was at the introductory workshop via paper and pencil. Participating students were randomly given vignette A and the other one-half received vignette B. Participants took approximately 10 minutes to describe the processes they would undertake to make a decision about the patient’s drug therapy. The second set of data was collected during a distance video conferencing session. Participants were given approximately 10 minutes to complete their response and invited to submit it electronically via the university’s learning management system. The vignettes were crossed-over so that participants received a different vignette pre- and post-intervention. Responses to demographic questions were also collected and included gender, ethnicity, time as practicing pharmacist and practice setting. A researcher (MA) who was not involved in the program anonymized the data by removing any identifying information from the responses and assigning a random identity code to maintain participant confidentiality and to ensure learners did not feel coerced to participate.

### 2.7. Data Analysis

**Figure 2:**
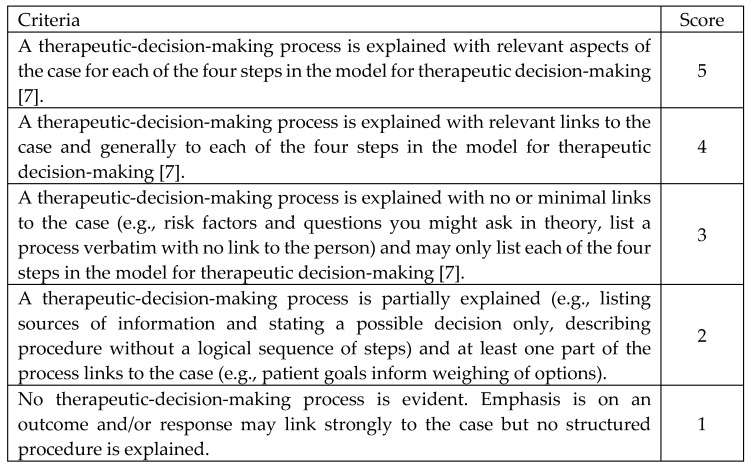
Scoring criteria for the vignettes. Note: The ideal response corresponds to score 5.

The vignette data were analyzed using the following process. All anonymized vignette responses collected pre- and post- teaching were randomized. A general inductive approach [19] was used to identify key features in common in participants’ written responses. An ideal response was written by the members of the research team with knowledge of clinical pharmacy practice (SD, DW), the learning outcomes and materials used in the module and informed by a model for decision making [7]. Descriptors were written for each point of a five-point scale (see Figure 2) with the ideal response representing a score of five. Each vignette response was compared to the descriptors by two of the four authors (SD, AP, KW, DW) who worked independently to assign a score. If scores differed, then the scoring procedure was repeated by another member of the scoring authors until agreement was reached. Exemplar responses were selected for each point along the scale to resolve any scoring disagreements. This process of anchoring to the descriptors and moderation to reach consensus about the score for each response ensured personal biases were addressed. 

The participants’ pre-intervention scores were compared to their post-intervention scores using a paired Wilcoxon matched-pairs signed rank test (2-tailed) in PRISM (v. 8.0.1, Graphpad, San Diego, CA, USA). The intention was to investigate if participants’ responses to the vignettes could usefully measure the development of therapeutic-decision-making skills of practicing pharmacists in the module. A sensitivity analysis was conducted to determine if the order that the students assessed each vignette made a difference to the scores. In this case, the pre-intervention scores between those students who completed vignette 1 were compared to those who completed vignette 2 using an unpaired Mann–Whitney test in PRISM. An alpha level of 0.05 was used for all statistical tests. Finally, the influence of years in practice on the difference between pre- and post-intervention scores was assessed using a regression analysis. The influence of practice setting (community or hospital) on the difference between pre- and post-intervention scores was compared using an unpaired Mann–Whitney test. 

## 3. Results

Twenty-eight practicing pharmacists (90% participation rate) consented to participate in this study and provided written responses to the pre-intervention and post-intervention vignettes. The demographics of these 28 participants generally reflected the distribution of the pharmacy profession in New Zealand for ethnicity (New Zealand European 19, Asian 6, Other 5, Māori 1, Pacific Island 1) and for gender (female 17, male 11) [20]. None of the participants had other pharmacy postgraduate qualifications or fellowships/ residencies (the latter are not offered in New Zealand). A summary of the demographics is presented in Table 1. 

**Figure 3:**
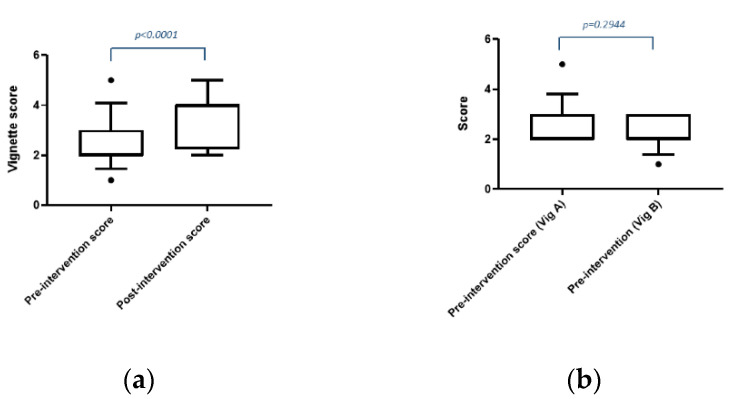
(**a**) Pre- and post-intervention scores. The boxes show the 25th and 75th percentiles of the data, the whiskers extend to include the 10th and 90th percentiles. Note that the median lines are the thicker edge of each box (pre-score median 2 and post-score median 4). The dots represent data points outside the 10th to 90th percentiles; (**b**) Pre-intervention scores for vignettes A and B. The boxes show the 25th and 75th percentiles of the data, the whiskers extend to include the 10th and 90th percentiles. Note that the median lines are the thicker edge of each box (median 2 for both vignette A and B). The dots represent data points outside the 10th to 90th percentiles.

The raw scores for participants’ responses are shown in Supplementary Materials and are summarised in Figure 3a. There was a median increase in score of 2 units on the five-point scale in the post-intervention scores compared to pre-intervention (*p* < 0.0001). Three students had lower post-intervention scores than the pre-intervention score and the scores for four students were unchanged. 

The results of the sensitivity analysis are presented in Figure 3b. There was no difference in the pre-intervention scores for students who completed vignette A compared to those who completed vignette B (*p* = 0.2944). These results suggest that the vignettes were comparable in terms of measuring decision-making skills and that the order that the students received the vignette did not influence the scores.

The influence of years in practice and practice setting on the difference in pre- and post-intervention scores are presented in Figure S1a,b. Years of practice was not found to influence the change in scores (R^2^ = 0.026, *p* = 0.92), although we note that five of the seven pharmacists who had no change in score or a lower post-intervention score had only 2 or 3 years of practice experience. Seven participants with similar practice experience recorded an increase in score post-intervention. Pharmacists practicing in a hospital setting had a larger median increase in score (2 units [range 0–3] in the post-intervention scores compared to community pharmacists (1 unit [range −1–3], *p* = 0.0426). We did not find a difference in practice experience between community (median 4 years) and hospital pharmacists (median 3 years). 

## 4. Discussion

The primary finding of this preliminary study is that we found a significant increase between pre- and post-interventional scores (2 units on the five-point scale, *p* < 0.0001) using a vignette to measure the development of therapeutic-decision-making skills by practicing pharmacists undertaking a university-based postgraduate clinical program at distance. The results of this study were cautiously interpreted to suggest that the participants’ responses to the vignettes are a reasonable measure of student learning. Therefore, we infer that the teaching and learning intervention successfully enabled the development of therapeutic-decision-making skills by practicing pharmacists. Some early-career pharmacists in our study had no change or a reduced score after our teaching. It is possible that this group may need more attention to develop these skills. The finding that pharmacists practicing in hospital settings showed greater gains than those in community contexts may warrant further investigation.

This study addresses a gap in pharmacy-education research that explores a possible teaching and learning approach to develop decision-making skills about drug therapy. By designing a module focused on skills required to make therapeutic decisions, practicing pharmacists are required to examine their own assumptions and habits of making decisions in their practice. Strengths that practising pharmacists bring to the course with them include information-gathering skills and a willingness to identify and defend the decisions they made. Skills corresponding to the reasoning and judgement steps of the model for therapeutic decision-making [7] appear to be focal areas of development during the course. 

This study also adds to the health-professional education field more generally. It has been proposed that making decisions about drug therapy in medical practice requires different cognitive skills than those used when making a diagnosis [10,11]. Diagnosis may require a deductive process where many potential options are refined to one. The patient is usually a bystander in the diagnostic process as the physician works through a process of objective and subjective data collection and assessment. Decisions about drug therapy are explicitly patient-centred because they focus on things like patient goals and patient preference. Therapy decisions require negotiation and several factors may need to be balanced to optimise care. In addition, a therapeutic decision may involve choosing between several viable options that are all reasonable in theory. In practice, the decision involves understanding which choice will be optimal for the individual patient involved, rather than optimal at a population level. The processes required to navigate these decisions, while accounting for complexities such as comorbidities, patient differences in demographics and preferences, are often tacit [9]. Our work suggests that these processes can be surfaced and are teachable as skills. According to Kirkpatrick’s Evaluation Model [21], our work shows demonstrated differences in learning (level 2). Future studies would need to be conducted to determine any changes in behavior or outcomes. 

Our decision to sample practicing pharmacists engaged in post-registration clinical qualifications may allow our results to be viewed as transferable to other continuing professional development contexts that have a several-weeks duration. Further work is needed to explore the development of therapeutic-decision-making skills of practicing pharmacists in other learning settings. To enhance the trustworthiness, robustness and credibility of our data analysis procedures, we used experienced pharmacy educators whose judgements were anchored to an ideal answer and they had to justify their scores until consensus was reached. Future work could explore how pre-registration pharmacists respond to the vignettes. We anticipate that their answers may differ from practicing pharmacists in the depth and breadth of knowledge about drug therapy. While the model for therapeutic decision-making used in this study emphasizes the cognitive aspects of decision making, it does so at the expense of emotive or cultural aspects. 

In summary, the findings from this study provide the first evidence of evaluation about teaching the process of therapeutic decision-making in a CPD setting. Further research is needed to determine if this method of teaching therapeutic decision-making is applicable to shorter learning-event durations and how this method might be used alongside or integrated with the PPCP framework.

## Figures and Tables

**Table 1:** Relevant demographic details of the participants.

Gender (n)	Practice Setting (n) ^1^	Years in Practice (Years) ^1^ Median (Range)	Ethnicity (n) ^2^
Female (17) Male (11)	Community (17) Hospital (9)	3.5 (2–35)	NZ European (19) Asian (6) Other (5) Māori (1) Pacific Island (1)

^1^ Data was only available for 26 participants. ^2^ Participants were able to self-identify as more than one ethnicity.

## Supplementary Materials

The following are available online at https://www.mdpi.com/2226-4787/8/2/83/s1. Figure S1: (**a**) Change in score pre- and post-intervention and years of pharmacy practice. The solid black line is the regression line. The years in practice was not found to predict the change in score (R^2^ = 0.026, *p* = 0.92). (**b**) Change in score pre- and post-intervention compared by practice site. Pharmacists practicing in a hospital setting had a larger median increase in score (2 units [range 0–3] in the post-intervention scores compared to community pharmacists (1 unit [range −1–3], *p* = 0.0426), Table S1: Raw scores by vignette types (A, B) and timing (pre-intervention, post-intervention) for participants (n = 28).
