# Supplementary material for: Measuring the Development of Therapeutic-Decision-Making Skills by Practicing Pharmacists Undertaking a University-Based Postgraduate Clinical Qualification at Distance
Source: Pharmacy (Basel). 2020 May 11;8(2):83. doi: 10.3390/pharmacy8020083 (PMC7355896; doi:10.3390/pharmacy8020083)
Supplement: Supplementary file 1 [file pharmacy-08-00083-s001.pdf]

Table 1. Raw scores by vignette types (A, B) and timing (pre-intervention, post-intervention) for participants (n = 28).

ID number	Pre-intervention vignette	Pre-intervention score	Post-intervention vignette	Post-intervention score	Difference between scores
1	A	3	B	4	1
2	A	2	B	2	0
3	B	3	A	4	1
4	A	2	B	2	0
5	B	2	A	2	0
6	A	2	B	3	1
7	A	2	B	4	2
8	B	2	A	4	2
9	A	3	B	4	1
10	A	3	B	2	-1
11	B	3	A	2	-1
12	B	2	A	4	2
13	B	2	A	5	3
14	B	2	A	4	2
16	B	1	A	2	1
17	A	2	B	3	1
18	B	3	A	5	2
19	B	2	A	2	0
20	A	3	B	5	2
21	B	3	A	5	2
22	A	2	B	4	2
23	A	2	B	4	2
24	B	2	A	3	1
26	A	3	B	5	2
27	B	2	A	4	2
28	A	3	B	3	0
30	A	5	B	4	-1
31	A	2	B	4	2
**Median**		2		4	2
**Range**		1-4		2-5	-1, 3
**Interquartile range**		2-3		2.25-5	0-2

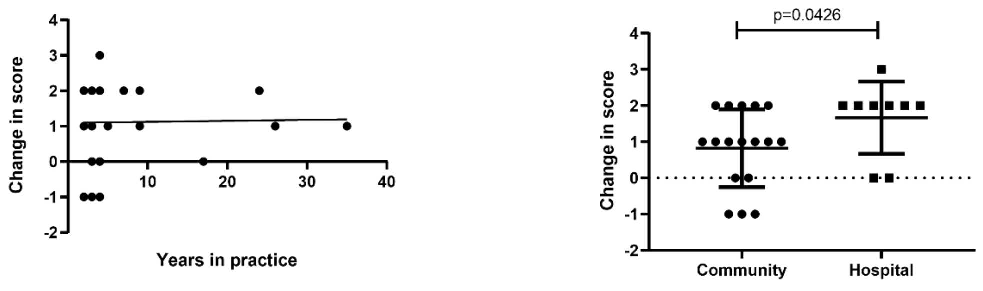

(a)

(b)

Figure S1. (**a**) Change in score pre- and post-intervention and years of pharmacy practice. The solid black line is the regression line. The years in practice was not found to predict the change in score (R^2=0.026, p=0.92). (**b**) Change in score pre- and post-intervention compared by practice site. Pharmacists practicing in a hospital setting had a larger median increase in score (2 units [range 0-3] in the post-intervention scores compared to community pharmacists (1 unit [range -1-3], p=0. 0426).
